# Characterization of brain microstructural abnormalities in cirrhotic patients without overt hepatic encephalopathy using diffusion kurtosis imaging

**DOI:** 10.1007/s11682-019-00141-4

**Published:** 2019-09-11

**Authors:** Qing Sun, Wenliang Fan, Yuan Liu, Yan Zou, Natalie Wiseman, Zhifeng Kou, Ping Han

**Affiliations:** 1 Department of Radiology, Union Hospital, Tongji Medical College, Huazhong University of Science and Technology, Wuhan, 430022 China; 2 Hubei Province Key Laboratory of Molecular Imaging, Wuhan, 430022 China; 3 Department of Biomedical Engineering, Department of Radiology, Wayne State University, Detroit, MI USA; 4 Department of Psychiatry and Behavioral Neurosciences, Wayne State University, Detroit, MI USA; 5 Department of Radiology, Wayne State University, Detroit, MI USA

**Keywords:** Minimal hepatic encephalopathy, Diffusion kurtosis imaging, Hepatitis B virus-related cirrhotic, Axial kurtosis, Axial diffusivity, White matter

## Abstract

Cirrhosis is a major public health concern. However, little is known about the neurobiological mechanisms underlying brain microstructure alterations in cirrhotic patients. The purpose of this prospective study was to investigate brain microstructural alterations in cirrhosis with or without minimal hepatic encephalopathy (MHE) and their relationship with patients’ neurocognitive performance and disease duration using voxel-based analysis of diffusion kurtosis imaging (DKI). DKI data were acquired from 30 cirrhotic patients with MHE, 31 patients without MHE (NMHE) and 59 healthy controls. All DKI-derived parametric maps were compared across the three groups to investigate their group differences. Correlation analyses were further performed to assess relationships between altered imaging parameters and clinical data. Voxel-based analysis of DKI data results showed that MHE/NMHE patients had increased radial diffusivity, axial diffusivity (AD) and mean diffusivity in addition to decreased axial kurtosis (AK) and fractional anisotropy of kurtosis in several regions. Compared to controls, these regions were primarily the cingulum, temporal and frontal cortices. The DKI metrics (i.e., AK and AD) were correlated with clinical variables in the two patient groups. In conclusion, DKI is useful for detecting brain microstructural abnormalities in MHE and NMHE patients. Abnormal DKI parameters suggest alterations in brain microstructural complexity in cirrhotic patients, which may contribute to the neurobiological basis of neurocognitive impairment. These results may provide additional information on the pathophysiology of cirrhosis.

## Introduction

Minimal hepatic encephalopathy (MHE) is a significant complication of cirrhosis with subclinical neurocognitive impairment. It is a major public health burden among cirrhotic patients (Bajaj et al. 2009). Patients with MHE usually display no identifiable or obvious clinical symptoms of overt hepatic encephalopathy (OHE), which is a severe neuropsychiatric complication of hepatic cirrhosis (Ferenci et al. 2002). However, MHE can impair patients’ quality of life and work productivity due to impairments in various neurocognitive functions, including memory, motor control, cognitive functions and psychomotor speed (Bajaj et al. 2009). MHE can also worsen over time (Bajaj et al. 2008) and progress to OHE (Gomez-Anson et al. 2015). Neuropsychological dysfunctions are common in cirrhotic patients before OHE occurs, as assessed by neurocognitive tests (e.g., psychometric hepatic encephalopathy score (PHES)). To date, neurocognitive impairments and motor control dysfunction have been reported in cirrhotic patients relating to the morphologic abnormalities induced by cirrhosis (Guevara et al. 2011; Chen et al. 2012). However, the neural microstructural alterations underlying a homogenous patient cohort with MHE or without OHE are still not fully understood.

The PHES test was recommended and validated as the preferential psychometric test score to evaluate neurocognitive impairments in many prior studies of cirrhosis (Duarte-Rojo et al. 2011; Lv et al. 2013a, b). The PHES consists of five neurocognitive tests designed to assess different aspects of cognitive and motor functions in cirrhosis patients, including motor speed and accuracy, attention, memory and visual function. Patients with MHE can be distinguished from patients without MHE or healthy participants by using the PHES test. However, the PHES is not sensitive enough to detect early neurological changes (Ferenci et al. 2002; Stewart et al. 2010), and the neurobiological mechanisms underlying these impairments are still unclear.

Recent advances in magnetic resonance imaging (MRI) such as diffusion tensor imaging (DTI) and diffusion kurtosis imaging (DKI) can provide more insight into the microstructural alterations caused by various diseases. Several previous studies using neuroimaging techniques reported abnormalities in patients with cirrhosis including low-grade edema and atrophy as patients developed MHE (Rovira et al. 2001, 2008; Guevara et al. 2011). Traditional DTI can measure water diffusion based on the assumption that water molecules displace in a Gaussian distribution (Basser and Jones 2002). DTI-derived parameters such as fractional anisotropy (FA) can assess the integrity of white matter tracts and specific DTI-derived parameter alterations may be markers for neurocognitive deficits in cirrhosis (Kumar et al. 2008; Chen et al. 2015). However, the tensor model for water diffusion in brain tissue limits the detection of abnormalities in gray matter and white matter regions with crossing fibers. Furthermore, water diffusion displacement is not actually Gaussian in biological tissue due to the presence of organelles and cell membranes (Wheeler-Kingshott and Cercignani 2009; Jensen and Helpern 2010; Abhinav et al. 2014). Therefore, a complete understanding of the microstructural changes in the brains of MHE patients based on DTI findings is limited.

As an extension of DTI, DKI was introduced to quantify the deviation of non-Gaussian water molecule diffusion in neural tissues (Narita et al. 2016). This method is thought to be a complementary technique to traditional DTI for a more comprehensive assessment of microstructural integrity, including in tissue with crossing fibers tracts (Chen et al. 2017). This has been shown to be more sensitive in detecting the microstructural changes associated with diseases than other diffusion tensor-based methods (Umesh et al. 2014). Quantitative metrics of DTI and DKI (e.g., mean kurtosis (MK), axial kurtosis (AK), and radial kurtosis (RK)) can be extracted from the analysis of DKI data to represent the diffusion of water molecules (Jensen and Helpern 2010). DKI has been successfully applied to investigate microstructural alterations in various diseases including Alzheimer’s disease (Gong et al. 2013) and stroke (Grinberg et al. 2014). Therefore, DKI may reveal new insights into the pathophysiology of neurocognitive alterations caused by cirrhosis.

Given this context, the aims of the present study were two-fold: i) to investigate microstructural alterations in hepatitis B virus-related cirrhosis (HBV-RC) patients with MHE and patients without MHE (NMHE) using DKI; and ii) to study the relationships between abnormal DKI parameters and the neurocognitive performance and disease duration in these patients. Our hypotheses were that i) DKI could evaluate microstructural changes in MHE and NMHE patients, and ii) DTI and DKI metrics are correlated with patients’ clinical and neurocognitive characteristics. Our goal is to enhance our understanding of the neurobiological characteristics of the neurocognitive alterations related to cirrhosis.

## Materials and methods

### Ethics

The study protocol was approved by the Medical Ethics Committee at Tongji Medical College of Huazhong University of Science and Technology. All subjects were informed of the purpose of the study and provided written consent before participating in accordance with Chinese legislation. After completion of the data collection, the data were renamed to remove any personally-identifying information pertaining to the subjects. Only these renamed data were analyzed for this report and all participating authors in this manuscript accessed only the renamed data.

### Study population and design

In total, 30 HBV-RC patients with MHE (MHE; mean age: 47.8 + 11.1 years, 23 males; right-handed), 31 patients with cirrhosis without MHE (mean age: 46.4 + 8.3 years, 27males; right-handed), and 59 age-, gender-, and education-matched healthy controls (mean age: 46.0 ± 9.9 years, 44 males; right-handed) were prospectively enrolled at the Union Hospital during the period between January 2016 and December 2016. All eligible healthy controls had no medical history of neurological diseases, liver diseases or any other severe organic disease. The clinical diagnosis of HBV-related cirrhosis was based on available clinical data from laboratory examinations and clinical diagnostic imaging results, including FibroTouch and abdominal computerized tomography with standard clinical practice guidelines (Oberti et al. 1997). The functional liver status of all patients was assessed by using Child–Pugh scores. The medical history of each participant was recorded. For controls, liver disease was ruled out by clinical and serologic analysis. All clinical diagnoses were made by our board-certified clinicians.

The following specific inclusion criteria were applied for the control group: (1) normal results for renal, cognitive and liver function, and no infection with any hepatitis or HIV; (2) absence of chronic liver or renal diseases, neurological or psychiatric diseases, or any other disease that can affect cognitive or motion function; (3) no abuse of alcohol or consumption of psychotropic drugs; (4) right-handedness; (5) normal participants’ cerebral and abdominal MRI results. For the patient group, inclusion criteria included: (1) HBV-related cirrhosis, but no other types of cirrhosis and OHE; (2) a normal ability to listen, read, write and speak, as well as the ability to cooperate with the doctors to complete all neurocognitive tests; (3) right-handedness; and (4) absence of severe renal, neurological or psychiatric diseases.

Exclusion criteria for all subjects included the presence of clinical manifestations of OHE or other neurological or psychiatric diseases in the past or present; taking drugs which may affect cognitive function; left-handedness; infection with other types of viral hepatitis or HIV; carcinoma; uncontrolled endocrine or metabolic disease (e.g., thyroid dysfunction) or alcoholism; a history of serious renal, respiratory and cardio-cerebrovascular impairments; or any obvious brain lesions (e.g., brain trauma, tumors or stroke) on conventional MRI. In our study, 105 patients met our inclusion criteria. 26 patients refused to participate, and 18 patients were not included due to their failure to complete the MRI examination process or poor MRI image quality.

### Neurocognitive assessment

A neuropsychologist blinded to health status administered the neuropsychological assessment, including the PHES, a battery of five tests (the number connection test-A (NCT-A), number connection test B (NCT-B), the digit-symbol test (DST), serial dotting test (SDT), line tracing test (LTT), and the Mini-Mental State Examination (MMSE), to all participants before their MRI scan. All participants completed the neurocognitive assessment. The MMSE test was used to assess the mental function of all participants. The PHES test battery for the diagnosis of MHE has been validated in many prior studies (Duarte-Rojo et al. 2011; Lv et al., 2013a, b). It was recommended as an important psychometric test tool to evaluate neurocognitive impairments (such as visual perception, attention and memory dysfunctions) in cirrhotic patients (Gomez-Anson et al. 2015; Lv et al., 2013a, b; Weissenborn 2008; Weissenborn et al. 2001). Higher test scores on the NCT-A, NCT-B, SDT, LTT indicate worse neurocognitive performance, while higher results of DST indicate better performance. MHE was diagnosed if cirrhotic patients’ PHES test scores were more than two standard deviations from the mean neurocognitive test performance of healthy subjects (Duarte-Rojo et al. 2011; Qi et al. 2013). Based on PHES test results, patients with cirrhosis were divided into the NMHE and MHE groups.

### Image acquisition

All MRI images were acquired on a GE Discovery 3.0 T MRI scanner 750 W (Grandview Blvd Waukesha, WI, USA). DKI experiments were performed using an echo-planar imaging diffusion sequence with an encoding scheme of 30 gradient directions. Other imaging parameters were: TR = 8000 ms, TE = minimum, FOV = 256 mm × 256 mm, matrix = 128 × 128, slice thickness = 4.0 mm, spacing = 0 mm, flip angle = 90°; three b-values (b = 0, 1000, 2000 s/mm^2^). The scan duration for DKI was 8 min and 16 s. For anatomical reference and image segmentation, a three dimensional brain volume imaging (3D BRAVO) sequence covering the whole brain was also acquired with the following parameters: TR = 9.1 ms, TI = 400 ms, TE = 3.5 ms, FOV = 256 mm × 256 mm, matrix = 256 × 256, 180 slices, slice thickness = 1.1 mm, scan duration = 4 min and 54 s. Additionally, axial fast spin echo T2-weighted fluid-attenuated inversion recovery (FLAIR) images (TR/TE = 12,000/120 ms, TI = 2200 ms, and matrix = 224 × 320), were also obtained to evaluate white and gray matter hyper-intensities in clinical diagnosis.

A visual inspection of DKI and 3D T1-weighted BRAVO images was performed to ensure the absence of obvious motion or other types of artifacts. Two radiologists independently reviewed the MRI scans; one radiologist had 10 years of experience, and the other had more than 30 years of experience.

### Image processing

First, the raw DKI data were corrected for eddy-current distortions and head motion using the FMRIB’s Diffusion Toolbox (FDT) toolkit available in FMRIB Software Library (FSL, Oxford, UK, http://www.fmrib.ox.ac.uk/fsl/) (Smith et al. 2004). Second, the DKI data were processed using Diffusional Kurtosis Estimator (DKE) version 2.6.0 (http://nitrc.org/projects/dke/) to generate parametric maps of DTI metrics, such as FA, mean diffusivity (MD), radial diffusivity (RD), axial diffusivity (AD), as well as DKI metrics (e.g., MK, RK, AK, KFA) of all participants. For data analysis, we adopted a voxel-based analysis (VBA), which is an unbiased approach of structural analysis and also an extension of voxel-based morphometry (Ashburner and Friston 2000). It has been widely used to investigate structural abnormalities across the entire brain in many neurological diseases, such as temporal lobe epilepsy (Kakeda and Korogi 2010) and cirrhosis with OHE. VBA enables a voxel-by-voxel comparison of unbiased diffusion-based parameters in the whole brain across subjects. Specifically, we applied Statistical Parametric Mapping (SPM8, http://www.fil.ion.ucl.ac.uk/spm/software/spm8/) to post-process these diffusion and kurtosis metrics. For the normalization: the b = 0 image of each subject was registered to the T1-weighted image using a 12-parameter (affine) linear transformation. The T1-weighted images were spatially normalized to the Montreal Neurologic Institute (MNI) (2.0 mm isotropic voxels) using the nonlinear registration module of the Automatic Registration Toolbox. The transformation matrices obtained from these previous steps were then applied to corresponding diffusion maps (FA, MD, RD and AD maps) and DKI maps (MK, RK, AK and KFA). Afterwards, every co-registered and normalized parametric map was smoothed with a full width at half maximum 6-mm Gaussian kernel. Finally, we evaluated the differences among the three groups regarding all DTI and DKI-derived parameters. The outcomes were visualized with the Data Processing and Analysis of Brain Imaging (DPABI) toolbox Viewer (http:// http://rfmri.org/dpabi).

### Statistical analysis

All statistical analyses for demographics were performed with the Statistical Package for the Social Sciences 21.0 for Windows (SPSS, Chicago, Illinois). Statistical analyses for demographic and clinical data were conducted using one-way analysis of variance (ANOVA), Chi-squared test, Student’s t test and Mann-Whitney tests, and the statistical thresholds were set at *p* < 0.05. All results are given as the means ± SD. To detect all DTI and DKI-derived parameters differences among the three groups, a one-way analysis of covariance (ANCOVA) and post hoc two-sample t-tests were performed with gender, age, and education as covariates, with corrections for a false discovery rate (FDR) applied at *p* < 0.05 with an extent threshold greater than 10 voxels. Brain regions showing group differences in the measured parameters compared to healthy subjects were defined as regions of interest (ROIs), and the mean values of these parameters of each ROI were extracted. We performed the Pearson correlation analysis between these extracted values and clinical variables (i.e., duration of disease and PHES scores) in the two patient groups using a significance threshold of *p* < 0.05.

## Results

### Demographic and clinical characteristics

The demographic, clinical and neuropsychological characteristics of the healthy subjects, MHE and NMHE patients are shown in Table 1. No significant differences were found in terms of age, education level and gender between any two of the three groups (p < 0.05). The performances of all subjects on the MMSE test were normal. Cirrhotic patients with MHE had significantly worse PHES test scores compared to healthy subjects (p < 0.05). There were no significant differences in PHES test scores between NMHE patients and healthy controls (*p* > 0.05).

**Table 1:** Demographic and clinical characteristics in MHE and NMHE patients with cirrhosis and normal controls

Demographic	MHE (*n* = 30)	NMHE (*n* = 31)	Control (*n* = 59)	*p* value
Gender (male/female)	23/7	27/4	44/15	*p* = 0.379
Age (years)	47.8 + 11.1	46.4 + 8.3	46.0 + 9.9	*p* = 0.714
Education level (years)	6.7 + 4.4	7.6 + 4.8	8.4 + 3.6	*p* = 0.177
Disease duration (years)	8.7 + 4.8	5.5 + 3.9	–	*p* = 0.007*
Child–Pugh’s class: A/B/C (n)	8/10/12	22/3/6	–	–
MMSE (scores)	27.5 + 1.4	27.7 + 1.3	28.1 + 1.3	*p* = 0.145
PHES (scores)	−7.6 + 2.9	0.3 + 1.0	0.1 + 1.5	*p* < 0.001*
DST (scores)	32.6 + 5.9	49.5 + 9.5	49.5 + 9.9	*p* = 0.000*
SDT (scores)	64.9 + 10.4	49.4 + 6.0	44.7 + 8.5	*p* = 0.000*
NCT-A (scores)	70.9 + 12.4	52.1 + 8.0	46.4 + 10.1	*p* = 0.000*
NCT-B (scores)	96.4 + 16.7	65.0 + 10.5	59.7 + 12.2	*p* = 0.000*
LTT (scores)	70.6 + 11.2	52.9 + 6.2	47.5 + 9.2	*p* = 0.000*

1) All values are displayed as mean ± standard deviation (SD). *Significant differences (*p* < 0.05)

2) The *p* values for gender distribution and disease duration were obtained by chi-square test and two-sample Student’s t test, respectively

3) The *p* values for age, education level and neuropsychological tests scores were obtained by one-way analysis of variance

4) Abbreviations: *MHE* Minimal hepatic encephalopathy, *NMHE* Without minimal hepatic encephalopathy, *MMSE* Mini-mental state examination, *PHES* Psychometric hepatic encephalopathy score, *DST* Digit-symbol test, *SDT* Serial dotting test, *NCT-A* The number connection test-A, *NCT-B* Number connection test B, *LTT* Line tracing test

### Voxel-based analysis of DKI data

**Fig. 1:**
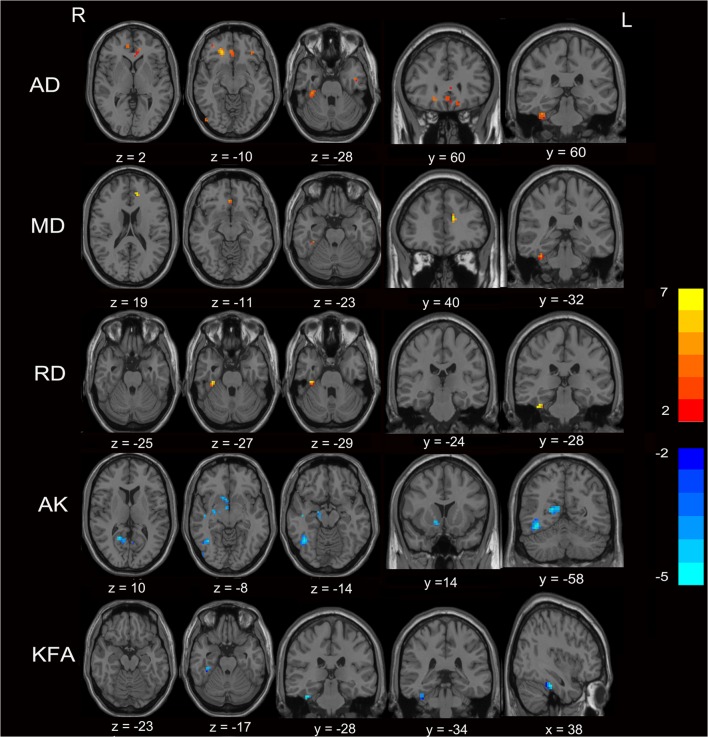
Comparison of diffusion and kurtosis metrics between MHE patients and healthy controls (*p* < 0.05, FDR corrected). Voxel-based analysis results showed regions with increased AD, MD, RD (in red and yellow) and areas with reduced AK and KFA (in blue) in MHE patients as compared to healthy subjects. All images are displayed in Montreal Neurological Institute space using neurological convention. The color bar reflects T values. Abbreviations: *MHE* Minimal hepatic encephalopathy, *AD* Axial diffusivity, *MD* Mean diffusivity, *RD* Radial diffusivity, *AK* Axial kurtosis, *KFA* Kurtosis of fractional anisotropy, *FDR* False discovery rate, *R* Right, *L* Left

**Fig. 2:**
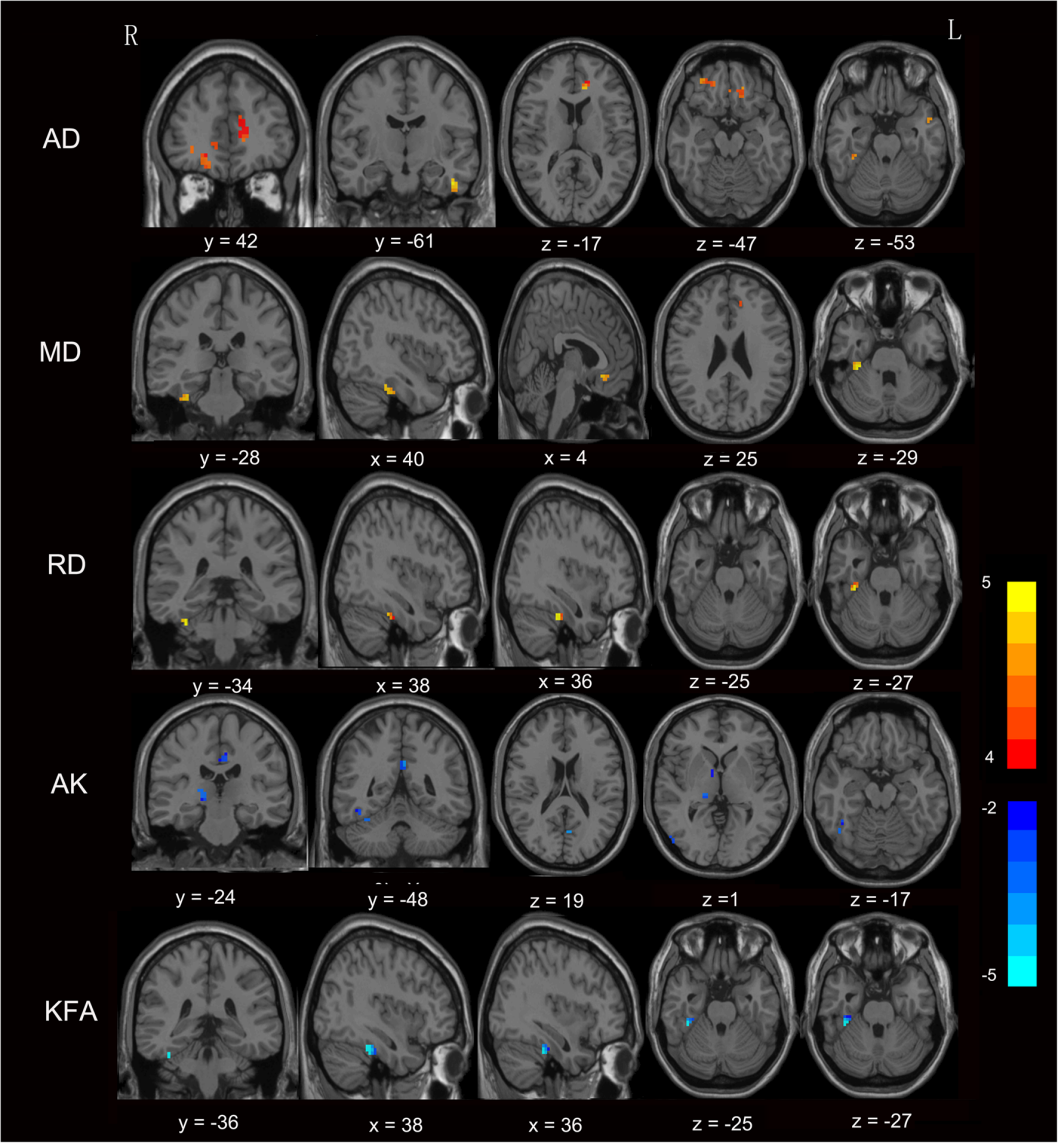
Comparison of diffusion and kurtosis metrics between NMHE patients with cirrhosis and control subjects (*p* < 0.05, FDR corrected). Voxel-based analysis results exhibited brain regions with increased AD, MD, RD (in red and yellow) and areas with decreased AK and KFA (in blue) in NMHE patients when compared with healthy subjects. The color bar reflects T values. Abbreviations: *NMHE* Without minimal hepatic encephalopathy, *AD* Axial diffusivity, *MD* Mean diffusivity, *RD* Radial diffusivity, *AK* Axial kurtosis, *KFA* Kurtosis of fractional anisotropy, *FDR* False discovery rate, *R* Right, *L* Left

Figure 1 and Table 2 show clusters with significant decreases in AK and KFA in patients with MHE, particularly in the fusiform, calcarine, temporal gyrus, hippocampus, caudate nucleus, thalamus and middle cingulum, as well as increased MD and AD in the fusiform gyrus, superior frontal gyrus and anterior cingulum (p < 0.05). Moreover, when compared with controls, NMHE patients showed AK, KFA reductions mainly in the cerebelum_4_5, olfactory, temporal, lingual gyrus, hippocampus, thalamus and posterior cingulum as well as AD, RD, MD increases in the anterior cingulum, temporal gyrus and cerebelum_6 (p < 0.05) (Fig. 2; Table 3). There were no significant findings in the FA, MK and RK values among the three groups (*p* > 0.05).

**Table 2:** Brain areas showing diffusion and kurtosis parameter differences between MHE patients and healthy controls

Diffusion parameters	Brain regions	Peak MNI, mm			T scores	*p* value
		X	Y	Z		
AK	Right fusiform gyrus	24	−27	−21	−4.06	0.008
	Right inferior temporal gyrus	45	−17	−18	−4.89	0.002
	Right hippocampus	18	−9	−12	−4.76	0.002
	Right middle temporal gyrus	54	−72	3	−3.96	0.010
	Right caudate nucleus	6	7	−5	−4.48	0.003
	Right thalamus	18	−24	0	−4.50	0.003
	Right calcarine	18	−54	12	−3.77	0.002
	Left middle cingulum	−3	−9	36	−5.06	0.002
KFA	Right fusiform gyrus	36	−30	−27	−6.06	0.000
MD	Right fusiform gyrus	36	−30	−27	4.82	0.003
	Right medial orbital parts of right superior frontal gyrus	3	33	−9	4.37	0.013
	Left anterior cingulum	−12	42	15	4.91	0.003
AD	Right fusiform gyrus	36	−30	−27	4.80	0.002
	Right inferior temporal gyrus	−42	−3	−30	4.63	0.003
	Right orbital parts of right superior frontal gyrus	21	36	−12	7.42	0.000
	Right inferior occipital gyrus	51	−79	−3	4.44	0.008
	Right medial orbital parts of right superior frontal gyrus	3	36	−12	4.37	0.002
	Left rectus gyrus	−12	36	−18	4.89	0.002
	Left orbital parts of left inferior frontal gyrus	−36	36	−6	5.16	0.006
	Left anterior cingulum	−12	42	12	5.14	0.002
RD	Right fusiform gyrus	36	−30	−27	4.80	0.002

Abbreviations: *MNI* Montreal neurological institute, *MHE* Minimal hepatic encephalopathy, *AK* Axial kurtosis, *KFA* Kurtosis of fractional anisotropy, *MD* Mean diffusivity, *AD* Axial diffusivity, *RD* Radial diffusivity, *R* Right. The statistical threshold was set at a false discovery rate–corrected *p* < 0.05, with an extent threshold greater than 10 voxels

**Table 3:** Brain regions showing diffusion and kurtosis parameter differences between NMHE and healthy controls

Diffusion parameters	Brain regions	Peak MNI, mm			T score	*p* value
		X	Y	Z		
AK	Cerebelum_4_5_R	33	−33	−30	−5.43	0.001
	Right inferior temporal gyrus	48	−60	−15	−4.40	0.010
	Right hippocampus	18	−9	−15	−3.40	0.042
	Right middle temporal gyrus	54	−69	3	−3.44	0.042
	Right Olfactory gyrus	3	9	−6	−3.78	0.001
	Right thalamus	18	−24	3	−4.50	0.020
	Right Lingual gyrus	12	−60	9	−3.90	0.018
	Left calcarine	−6	−63	9	−3.96	0.018
	Left posterior cingulum	−3	−48	30	−3.91	0.018
KFA	Right fusiform gyrus	36	−30	−24	−4.69	0.001
MD	Cerebelum_4_5_R	36	−30	−30	5.69	0.000
	Right medial orbital parts of superior frontal gyrus	0	30	−9	5.05	0.000
AD	Left inferior temporal gyrus	−45	−9	−27	5.50	0.000
	Left orbital parts of superior frontal gyrus	−15	39	−18	4.02	0.015
	Left middle temporal gyrus	−51	6	−27	4.69	0.002
	Left anterior cingulum	−6	36	3	5.67	0.000
	Cerebelum_6_R	36	−30	−30	5.73	0.001
	Right medial orbital parts of superior frontal gyrus	14	42	0	3.04	0.003
	Right orbital parts of superior frontal gyrus	21	36	−12	4.66	0.002
RD	Cerebelum_6_R	36	−30	−30	5.64	0.000

Abbreviations: *MNI* Montreal neurological institute, *MHE* Minimal hepatic encephalopathy, *AK* Axial kurtosis, *KFA* Kurtosis of fractional anisotropy, *MD* Mean diffusivity, *AD* Axial diffusivity, *RD* Radial diffusivity, *R* Right. The statistical threshold was set at a false discovery rate–corrected *p* < 0.05, with an extent threshold greater than 10 voxels

### Correlation between the DKI parameters and clinical and neuropsychological data

**Fig. 3:**
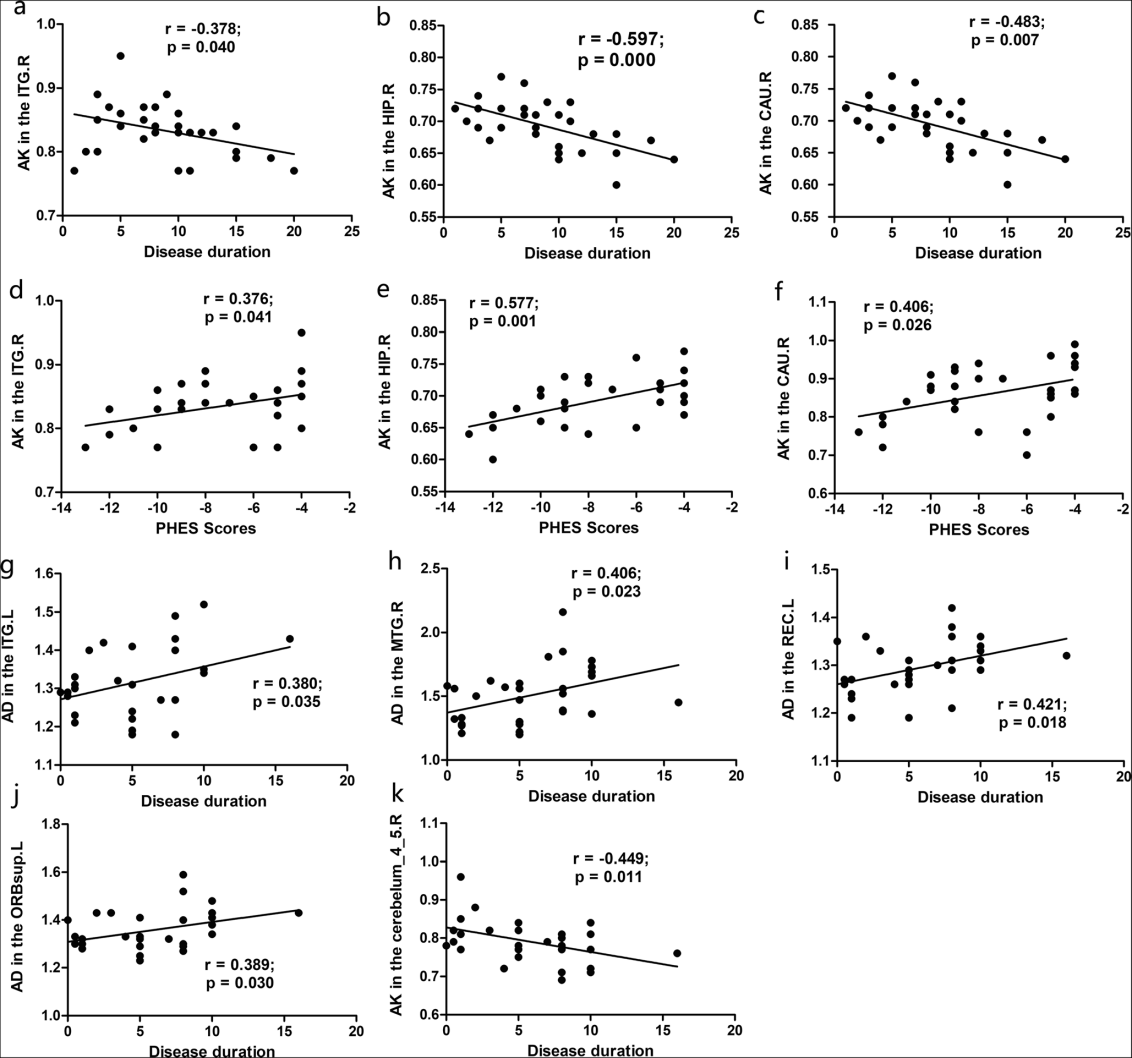
Correlations between diffusion and kurtosis parameters and clinical variables. For MHE patients, disease duration was negatively correlated with the decreased AK in the ITG.R (**a**), HIP.R (**b**), CAU.R (**c**); and PHES test scores were positively correlated with AK reductions in the ITG.R (**d**), HIP.R (**e**), CAU.R (**f**). As for the NMHE group, disease duration was positively correlated with increased AD in the ITG.L (**g**), MTG.L (**h**), REC.L (**i**), ORBsup.L (**j**) and negatively related with decreased AK in the cerebellum_4_5_R (**k**). Abbreviations: *MHE* Minimal hepatic encephalopathy, *NMHE* Without minimal hepatic encephalopathy, *AK* Axial kurtosis, *AD* Axial diffusivity, *ITG* Inferior temporal gyrus, *HIP* Hippocampus, *CAU* Caudate nucleus, *MTG* Middle temporal gyrus, *REC* Rectus gyrus, *ORBsup* Orbital parts of superior frontal gyrus, *R* Right, *L* Left

In the correlation analyses in MHE patients (Fig. 3), disease duration is negatively correlated with AK values in the right inferior temporal gyrus (r = −0.378, *p* = 0.040; Fig. 3(a)), right hippocampus (r = −0.597, *p* = 0.000; Fig. 3(b)) and right caudate nucleus (r = −0.483, *p* = 0.007; Fig. 3(c)); and PHES scores are positively correlated with AK values in the right inferior temporal gyrus (r = 0.376, *p* = 0.041; Fig. 3(d)), right hippocampus (r = 0.577, *p* = 0.001; Fig. 3(e)) and right caudate nucleus (r = 0.406, *p* = 0.026; Fig. 3(f)). For the NMHE patients, there are positive correlations between disease duration and AD values within the left inferior temporal gyrus (r = 0.380, *p* = 0.035; Fig. 3(g)), left middle temporal gyrus (r = 0.406, *p* = 0.023; Fig. 3(h)), left rectus gyrus (r = 0.421, *p* = 0.018; Fig. 3(i)) and left orbital parts of superior frontal gyrus (ORBsup.L) (r = 0.389, *p* = 0.030; Fig. 3(j)) as well as negative correlation with AK values in the right cerebelum_4_5 (r = −0.449, *p* = 0.011; Fig. 3(k)). PHES test scores are correlated with decreased AK values in the right cerebelum_4_5 (r = 0.499, *p* = 0.004) and increased AD in the ORBsup.L (r = −0.437, *p* = 0.014) in the NMHE group. There is no significant correlation between clinical variables and other diffusion metrics in either patient group.

## Discussions

In the current study, we investigated the whole-brain microstructural alterations in a cohort of HBV-RC patients with and without MHE using VBA of DKI data. The main findings of this study were as follows: (1) Regions of increases in AD, RD, MD and reductions in AK and KFA, predominately in the frontal, temporal and limbic areas (i.e., the cingulum), were observed in both HBV-RC subgroups (MHE and NMHE) compared to healthy controls. This suggests abnormalities in brain microstructure in both MHE and NMHE patients; (2) Correlations were detected between DTI/DKI parameters in significant brain clusters and clinical variances, such as neurocognitive performances (e.g., PHES test scores) and disease duration in MHE and NMHE patients.

### Alterations in axial and radial directional diffusion/kurtosis parameters of water

The AK and AD-directional diffusivity parameters can provide information regarding water diffusion characteristics along the primary diffusion direction. They are frequently used to characterize neural tissue microstructure (i.e., the integrity of axons) (Cheung et al. 2009; Gao et al. 2009). MHE and NMHE patients had reductions in AK in the left middle cingulum and left posterior cingulum respectively, suggesting declined microstructural complexity in MHE/NMHE. Increased AD in the left anterior cingulum indicated impaired microstructural integrity in cirrhosis with MHE/NMHE, possibly as a result of reduced axonal caliber allowing faster water molecule movement parallel to axons. Furthermore, increased RD, which is a reflection of myelination (Gao et al. 2009), was found in the right fusiform gyrus of MHE patients and cerebelum_6_R of NMHE patients, respectively. Several causes for alterations in the aforementioned diffusion parameters may be due to demyelination, hypomyelination, axonal damage or aberrant cytoarchitecture leading to increases in cerebrospinal fluid in brain (Song et al. 2003).

Our VBA of DKI results are in agreement with previous studies reporting significant abnormalities in gray and white matter using conventional DTI, especially in the association cortices and limbic regions (Ishihara et al. 2013; Kale et al. 2006). Lv et al., also reported abnormal gray matter structural networks in patients without OHE, such as frontal and temporal regions, using 3D T1-weighted images (Lv et al. 2015). Some previous studies have reported that MHE patients had poor driving performance, which led to an increase in the incidence of motor vehicle crashes (Bajaj et al. 2008, 2009). The frontal and temporal cortices are known to be involved in executive function, which is relevant to the skill of driving. Our results of decreased AK in the temporal gyrus and increased AD in the superior frontal gyrus might be related to executive dysfunction in MHE patients.

### Quantitative measurements of FA in MHE/NMHE patients

Consistent with previous studies (Kale et al. 2006; Kumar et al. 2008), we did not detect any significant changes in FA values between the two patient groups. FA is a quantitative index of anisotropy, and its changes are associated with three eigenvalues i.e., λ1、 λ2、 λ3. In brain DKI imaging, λ1 represents the diffusion coefficient along the direction of the white matter fiber, that is the axial diffusion coefficient AD. The term “Radial Diffusivity” refers to the mean of the λ2 and λ3. If diffusivity changes along both axial and radial directions occur symmetrically, no observable alterations would be found in FA (Acosta-Cabronero et al. 2010), thereby providing one possible reason for the unchanged FA in our study. Moreover, it is reported that FA may not accurately describe the microstructural alterations in brain regions with complex fiber configurations (Jensen and Helpern 2010), possibly explaining our failure to observe FA change. This neuroimaging study of cirrhosis may also be limited by the relatively small sample size. However, significant differences after corrections for multiple comparisons were found in the voxel-based analyses of DKI between groups. Future studies with larger sample size are needed to confirm and better delineate the neuroimaging results.

### Increased MD reflects low-grade cerebral edema in MHE and NMHE patients

The MD, an index of water movement across cell membranes, is an average of the major diffusion directions. Increased MD suggests increases in extracellular fluid. Similar to some studies previously reported (Ishihara et al. 2013; Kumar et al. 2008), our results of increased MD in the ORBsupmed.R and left anterior cingulum of MHE/NMHE patients compared to controls suggests a role of brain edema in cirrhotic patients. Previous neuroimaging and animal studies have reported low-grade cerebral edema in cirrhotic patients with MHE/NMHE (Assaf and Pasternak 2008; Jover et al. 2006; Kale et al. 2006; Kumar et al. 2008; Sugimoto et al. 2008). Glutamine plays an important role in cellular osmoregulatory in the brain, and astrocytes can detoxify ammonia to glutamine via glutamine synthetase. Kale et al., indicated that the extracellular migration of osmolytes, such as glutamine, during cellular osmoregulation may lead to increased accumulation of extracellular fluid, accounting for the increase in MD in patients with cirrhosis with OHE. Moreover, a significant increase in MD in NMHE patients compared with controls indicates that there is detectable brain edema using DKI even before the appearance of alterations on clinical and neurocognitive tests. This alteration is analogous to changes in myo-inositol (mI), choline (Cho), and Glx (glutamate) demonstrated with MR spectroscopy in patients without OHE. In summary, our results for MD indicate that DKI may be useful for detecting patients with MHE/NMHE, and further support the theory of the role of low-level cerebral edema in cirrhosis patients. This is consistent with prior studies using diffusion MRI (Rivera-Mancia et al. 2012; Sugimoto et al. 2008).

### Correlations between imaging parameters and clinical variables

Another important finding were the correlations between clinical variance and diffusion and kurtosis parameters in the frontal and temporal areas, hippocampus, caudate nucleus, and cerebellum, which were consistent with a previous diffusion study (Chen et al. 2017). The PHES test evaluates various neurocognitive domains, such as visual perception, executive function, attention and memory functioning in cirrhotic patients (Weissenborn 2008). For MHE patients, there were positive correlations between the reductions in AK in the right inferior temporal gyrus, hippocampus, caudate nucleus and PHES test scores. This result is similar to one previous study (Chen et al. 2017). The inferior temporal gyrus is responsible for visual, language and memory functions, and the hippocampus and caudate nucleus are involved in the memory processing and executive function, respectively (Eichenbaum 2017; Macfarlane et al. 2013). Therefore, our correlation analysis results are reasonable and indicate local microstructural alterations in those regions that may reflect clinical manifestations in MHE. In addition, we found correlations between decreased AK in the right inferior temporal gyrus, hippocampus and caudate nucleus of MHE patients and disease duration, which suggests that AK in these regions may reflect the progress of the disease. Finally, in the NMHE group, there are positive correlations between disease duration and increased AD within the language and cognitive function-related cortices, along with decreased AK in the cerebellum. This suggests changes in brain microstructure may have a cumulative effect of time as the disease progresses, so early clinical intervention are very crucial for the treatment of cirrhosis.

## Limitations

Several limitations of the present study should be noted. First, the disease etiology of patients in this study was limited to hepatitis B virus-related cirrhosis, so our results should be interpreted with caution when applied to cirrhosis patients with other etiologies, such as primary biliary cirrhosis. Second, the DKI voxel size is relatively large and non-isotropic, which may result in low sensitivity. Finally, our study is cross-sectional, and prospective longitudinal studies with large samples should be performed to further characterize these diffusion abnormalities in the future.

## Conclusions

In summary, our voxel-based analysis of DKI demonstrated microstructural abnormalities in the frontal, temporal areas and cingulum in MHE and NMHE patients with cirrhosis. DKI can provide additional information regarding the microstructural alterations in cirrhotic patients that account for their neurocognitive performance.

## Abbreviations

MHE: Minimal hepatic encephalopathy
DKI: Diffusion kurtosis imaging
HBV-RC: Hepatitis B virus-related cirrhosis
AK: Axial kurtosis
AD: Axial diffusivity
